# Thrombotic Microangiopathy Associated with Pazopanib in a Kidney Transplant Recipient

**DOI:** 10.15586/jkcvhl.v8i1.161

**Published:** 2021-03-24

**Authors:** Shabana Kalla, Robert J. Ellis, Scott B. Campbell, Brian Doucet, Nicole Isbel, Bibiana Tie, Dev Jegatheesan

**Affiliations:** 1 Department of Nephrology, Princess Alexandra Hospital, Brisbane, Queensland, Australia;; 2 Faculty of Medicine, University of Queensland, Brisbane, Queensland, Australia;; 3 Department of Anatomical Pathology, Princess Alexandra Hospital, Brisbane, Queensland, Australia

**Keywords:** kidney transplant, pazopanib, thrombotic microangiopathy, vascular endothelial growth factor inhibitors

## Abstract

Thrombotic microangiopathy (TMA) is characterised by abnormalities in the walls of arterioles and capillaries, precipitated by hereditary or acquired characteristics, and culminating in microvascular thrombosis because of dysregulated complement activity. A number of drugs can precipitate TMA, including vascular endothelial growth factor (VEGF) inhibitors, because of their effects on endothelial repair. Pazopanib is a VEGF inhibitor used for the treatment of renal cell carcinoma (RCC); it is uncommonly associated with TMA. A 52-year-old male, 5 years post his second kidney transplant secondary to immunoglobulin (Ig) A nephropathy, presented with hypertension, fluid overload, and worsening graft function (peak creatinine 275 µmol/L, baseline 130–160 µmol/L) and nephrotic range proteinuria 2 months after commencing pazopanib for metastatic RCC. His maintenance immunosuppression included ciclosporin, mycophenolate, and prednisolone. Haematological parameters were unremarkable. Allograft biopsy demonstrated glomerular and arteriolar changes consistent with chronic active TMA, with overlying features of borderline cellular rejection. He was treated with intravenous methylprednisolone 250 mg for 3 days and commenced on irbesartan 75 mg daily. Drug-induced TMA from pazopanib was suspected, particularly given the documented association with other tyrosine kinase inhibitors (TKIs). In consultation with his medical oncologist, pazopanib was ceased, and an alternate TKI cabozantinib was commenced. Serum creatinine remained <200 µmol/L 3 months after admission. This is the first reported biopsy-proven case of TMA attributed to pazopanib in a kidney transplant recipient. With increasing clinical indications for and availability of TKIs, clinicians need to be aware of their association with TMA events in kidney transplant recipients, who are already susceptible to TMA due to abnormal vasculature, infectious triggers, ischaemia-reperfusion injury, and use of calcineurin inhibitor.

## Introduction

Thrombotic microangiopathy (TMA) is characterised by abnormalities in the walls of arterioles and capillaries, culminating in microvascular thrombosis. Primary TMA syndromes are divided into hereditary, because of ADAMTS13 deficiency, or mutations affecting complement, metabolic, or coagulation pathways; and acquired, because of autoantibody inhibition of ADAMTS13, Shiga toxaemia, drug-mediated (either toxicity or immune reaction), and complement-mediated due to antibody inhibition of factor H activity (1).

The disease is usually characterised by microangiopathic haemolytic anaemia, which is associated with thrombocytopenia (2). The pathophysiology involves platelet consumption through the formation of intravascular thrombi. Erythrocytes become fragmented as they traverse the microthrombi. This is manifested as schistocytes on blood film and evidence of non-autoimmune intravascular haemolysis, with raised bilirubin, lactate dehydrogenase and reticulocyte count; low haptoglobin; and a negative Coombs test (2).

Drug-induced TMA (DITMA) involves either a complement-mediated process with the formation of drug-dependent antibodies to platelets, or the drug itself causing direct endothelial toxicity. Herein we report a case of TMA most likely induced by pazopanib toxicity, caused by direct inhibition of vascular endothelial growth factor (VEGF).

## Case Report

A 52 year old male kidney transplant recipient who developed kidney failure secondary to immunoglobulin (Ig) A nephropathy, who also had a history of metastatic clear cell renal cell carcinoma (ccRCC), on pazopanib, presented with acute kidney injury, nephrotic range proteinuria, hypertension, and fluid overload.

The patient initially received a deceased donor kidney transplant in 2001, with subsequent loss of the first allograft in 2014 because of chronic rejection, requiring haemodialysis in early 2015 until transplantation with a living related donor kidney later that year (five mismatches, unsensitised). The course of this second graft was immunologically uncomplicated with no episodes of rejection, and a stable creatinine of 130–160 µmol/L was maintained until the beginning of 2020. At the time of presentation, his immunosuppression regimen consisted of prednisolone 7 mg daily, ciclosporin 125 mg mane (morning)/100 mg nocte (night), trough level consistently <200 µg/L, and mycophenolate mofetil 1000 mg twice daily.

In 2018, a 155mm exophytic tumour of the right native kidney was identified following an episode of macroscopic haematuria, along with two suspicious pulmonary lesions (cT3a, N0, M1). The patient underwent cytoreductive (radical) nephrectomy and ccRCC was seen on histology. After 12 months of surveillance, marginal enlargement of pulmonary lesions was seen, and a new hyperdense exophytic soft tissue mass was seen at the head of the pancreas, which was thought to represent metastatic disease. Surveillance was continued while asymptomatic; however, the next few months were complicated by worsening liver function tests requiring endoscopic retrograde cholangiopancreatography (ERCP). New cerebellar metastases were also identified and managed with a posterior fossa craniotomy and short course of stereotactic radiotherapy.

Pazopanib was commenced post-radiotherapy, but this was interrupted after 2 months of treatment because of biliary sepsis and the need for a repeat ERCP. He recommenced pazopanib in late 2019, at which time baseline creatinine was stable at 131 µmol/L.

Over the next 2 months, the patient’s creatinine began to drift up, culminating in a hospital admission for hypertension (systolic blood pressure >180 mmHg on home sphygmomanometer) and peripheral oedema. He was haemodynamically stable at presentation, with a blood pressure of 156/98 mmHg (baseline systolic blood pressure <120 mmHg), and serum creatinine of 218 µmol/L.

Ultrasound of the transplant kidney was unremarkable, including normal intrarenal Dopplers and a patent renal vein. Urine microscopy was normal; however, he had nephrotic range proteinuria, with a urine protein to creatinine ratio of 745 g/mol. He was initially managed with furosemide. Nephrotoxins were withheld, including pazopanib.

**Figure 1:**
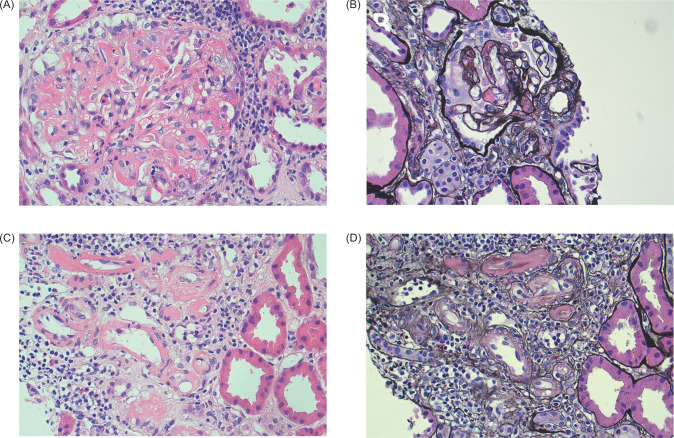
Light micrographs of kidney transplant biopsy. (A) Glomerulus with intracapillary fibrin, red cell fragments, and karryorhectic debris (hematoxylin and eosin × 40). (B) Segmentally sclerosed glomerulus with double contouring and fibrin (silver × 40). (C and D) Arterioles with fibrin, and focal onion skinning with myxoid intima, red cell fragments, and karyorrhexis (C: hematoxylin and eosin × 40; D: silver × 40).

**Figure 2:**
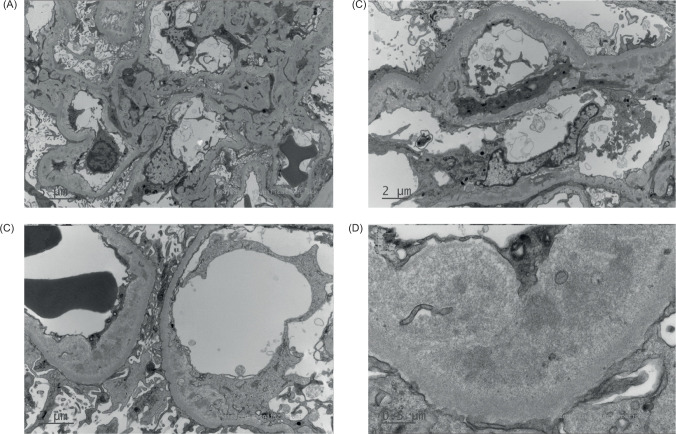
Electron micrographs of kidney transplant biopsy. (A) Mesangial expansion by increased mesangiocytic processes and electron dense deposits. (B and C) Subendothelial lucent widening with loss of endothelial fenestrations, mesangial interpositioning, and intramembranous electron-dense deposits. (D) Higher power view of deposits showing vaguely fibrillary substructure.

Owing to lack of improvement in his kidney function, he had an allograft biopsy on the third day three of admission, which demonstrated glomerular and arteriolar changes of chronic active TMA (Figure 1). Eight glomeruli and one small artery were sampled. There was one globally sclerosed glomerulus and four showing segmental sclerosis with occasional double contours. Most of the remaining glomeruli showed changes of thrombotic microangiopathy, including intracapillary fibrin deposition, karyorrhectic debris, and red cell fragments. Fibrin was also identified within thickened arteriolar intima, confirmed on special staining. Some corticomedullary arterioles demonstrated focal onion skinning with myxoid intima, fibrinoid change, karyorrhectic debris, and red cell fragments. No arterial involvement was seen. Electron microscopy demonstrated thickened glomerular basement membranes with prominent subendothelial lucent widening, loss of endothelial fenestrations, and overlying podocyte effacement. Widespread mesangial, paramesangial, intramembranous, and subendothelial electron dense deposits with a vaguely fibrillary substructure were also noted (Figure 2). Immunofluorescence demonstrated granular and globular subendothelial IgM, C3, and lambda, and specks of IgG, IgA, C1q, and kappa within sclerosed segments.

Chronic changes included 10–20% tubulointerstitial scarring, 50% segmental glomerulosclerosis, and 12.5% global glomerulosclerosis (ci1, ct1, cg0). Borderline T-cell rejection (t2, i0, ti1) and acute tubular necrosis were also noted on biopsy. There were no features that would indicate antibody-mediated rejection, and donor-specific antibodies (which can precipitate TMA) were not present.

Following the results of the biopsy, the patient was started on intravenous methylprednisolone 250 mg for 3 days to proactively manage any T-cell rejection. Serum creatinine peaked at 275 µmol/L on the fourth day of admission. Lactate dehydrogenase was mildly elevated. There were no haematological manifestations of TMA (Table 1), including absent schistocytes on film, normal platelet count, and normal bilirubin. Serum haptoglobin was elevated, which is inconsistent with TMA; however, elevated haptoglobin has been reported in patients with RCC, which may explain this finding (3). ADAMTS13 activity was normal. Shiga toxin was not tested for in the absence of diarrhoea; a Coombs test was not done in the absence of overt haemolysis. TMA was attributed to pazopanib as a diagnosis of exclusion. Atypical haemolytic uraemic syndrome (HUS) was deemed less likely, given the absence of haematological manifestations of TMA.

**Table 1:** Pertinent investigations on admission and discharge.

Investigation	Admission	Discharge
Haemoglobin	115 g/L	121 g/L
Platelet Count	172 ×10^9^/L	238 ×10^9^/L
Creatinine	218 µmol/L	244 µmol/L
Total Bilirubin	9 µmol/L	9 µmol/L
Lactate Dehydrogenase	393 unit/L	312 unit/L
Haptoglobin	—	2.89 g/L
ADAMTS13 Activity	—	0.91 IU/mL
Urine ACR	570 g/mol	—
Urine PCR	745 g/mol	—

Follow-up results were from 2 months post-discharge. ACR: albumin–creatinine ratio; PCR: protein–creatinine ratio.

**Figure 3:**
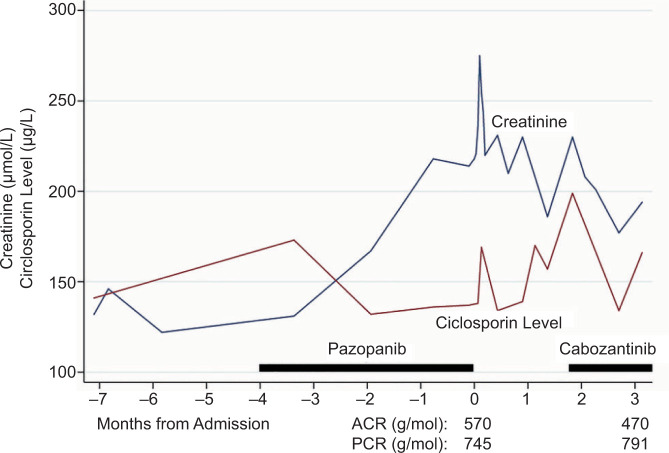
Serum creatinine and trough ciclosporin levels compared against time from admission, and commencement of both pazopanib and cabozantinib.

He was commenced on irbesartan 75 mg and discharged with a plan for outpatient optimisation of blood pressure. Eculizumab was considered; however, as DITMA was more likely than atypical HUS, and because serum creatinine continued to downtrend (Figure 3), eculizumab was not pursued. A shared decision was made between the patient and the nephrology and oncology teams to discontinue pazopanib, despite its efficacy in this case, and begin a trial of cabozantinib, a different class of multi-tyrosine kinase inhibitor with good effectiveness in metastatic ccRCC (4). This decision was made due to the patient’s prioritisation of maintaining allograft function balanced against oncological control, which was based on the considerations that he still operated his own business and lived in a rural area. Careful monitoring of kidney function was needed, as TMA has also been reported in association with cabozantinib (5). Three months following discharge, serum creatinine dropped below 200 µmol/L; however, the patient continued to experience nephrotic range proteinuria (Figure 3).

Written informed consent was provided by the patient to publish this case report.

## Discussion

We report a case of *de novo* kidney-limited TMA in a kidney transplant recipient, presumed secondary to pazopanib, prescribed for the treatment of metastatic ccRCC. This report is only the second biopsy-confirmed diagnosis of TMA associated with pazopanib, and the only biopsy-confirmed case of VEGF inhibitor-associated TMA in a kidney transplant recipient of which the authors are aware. We propose that TMA developed due to the anti-VEGF effects of pazopanib, and was predisposed by other transplant-related factors, including chronic use of calcineurin inhibitors.

Thrombotic microangiopathy is a well-known class effect of anti-VEGF agents, and there are numerous reports of TMA associated with the use of bevacizumab, sunitinib, sorafenib, and afibercept (6–8). The most likely mechanism of injury is direct VEGF inhibition, which, in the setting of high glomerular shear stress, perpetuates endothelial damage, and is conducive to the development of TMA (9, 10). Despite this theoretical risk, TMA is rarely reported in association with pazopanib, although proteinuria, hypertension, and thrombocytopenia are frequently observed without TMA in patients taking pazopanib (11–15). In these cases, proteinuria is most likely driven by podocyte effacement and exacerbated by concomitant hypertension (16).

Thrombotic microangiopathy is identified as a potential side effect of pazopanib by its manufacturers (17), and has been reported in trials combining pazopanib with bevacizumab (18); however, to the best of the authors’ knowledge, there are only two published case reports wherein pazopanib has been linked to TMA. The first case was of a 31-year-old male who had been managed with pazopanib for 8 months for rhabdomyosarcoma and presented with nephrotic syndrome, and was found to have endothelial damage suggestive of TMA on kidney biopsy, without systemic manifestations. In this case, pazopanib was suggested as the primary cause of TMA, and full recovery was seen following drug cessation (19). The second case was of a 76-year-old male, who was managed with pazopanib for 4 months following RCC diagnosis; he presented with acute kidney injury, hyperbilirubinaemia, and schistocytes on film, and was diagnosed with TMA on clinical grounds. This patient had low ADAMTS13 activity, which was probably the driving mechanism, rather than VEGF inhibition; enzyme activity increased following cessation of pazopanib and 1 week of plasmapheresis (20).

Given that the present case report was in a kidney transplant recipient, a number of additional considerations were to be made if attributing TMA to pazopanib. In absolute terms, *de novo* TMA occurs uncommonly in the post-transplant period; its incidence has been reported as 0.8% in 15,870 kidney transplant recipients, transplanted between 1998 and 2000, in a retrospective analysis of the United States Renal Data System (21). Despite this, TMA is also substantially more common in allograft than native kidneys, predisposed by abnormal vasculature, ischaemic injury, and frequent infectious triggers, and *de novo* TMA was reported in 2.8–13.8% of transplant biopsies across a number of studies (22). Notwithstanding, a majority of these cases were presumedly related to anti-rejection drugs, caused by both calcineurin inhibitors, through endothelial damage and direct effects that promote platelet aggregation (23), and mammalian target of rapamycin (mTOR) inhibitors, which have some anti-VEGF properties and cause lower renovascular and podocyte expression of VEGF (24). As the patient in the present case report was taking ciclosporin as part of his immunosuppression regimen, it is impossible to rule out that the presentation was solely due to the effects of ciclosporin. However, this hypothesis was clinically less likely, given the patient had been maintained on ciclosporin for many years with all recorded trough levels below 200 µg/L in the months leading up to TMA diagnosis, and because improvement of kidney function following cessation of pazopanib was seen despite ongoing use of ciclosporin. Furthermore, as pazopanib was started relatively recently, this supports the presumption that it was involved in the pathogenesis of TMA. The fact that TMA was limited to the kidney is also supportive that it developed secondary to VEGF inhibition, as this presentation is typical of patients who develop DITMA in association with this class of medication (25).

An interesting finding in this biopsy was the presence of widespread mesangial, paramesangial, intramembranous, and subendothelial electron dense deposits with vaguely fibrillary substructure. Similar electron-dense deposits have been reported in association with bevacizumab, attributed to serum protein leakage in the context of VEGF inhibition increasing endothelial permeability (26). A comparable mechanism could be involved in this case. The architecture of these deposits could be consistent with diabetic fibrillosis, but this is less likely because the patient always had good glycaemic control. Cryoglobulin deposition was another differential diagnosis. Although this hadn’t been specifically investigated, it is less likely given the clinical course.

A feasible consideration in this case is that multiple “hits” contributed to the development of TMA (27), whereby endothelial damage caused by ciclosporin was perpetuated by VEGF inhibition by pazopanib, leading to unchecked complement activation and platelet aggregation without endothelial recovery, over time causing the development of chronic TMA-like changes that were seen on biopsy. Although reports of TMA in transplant recipients taking both calcineurin and direct VEGF inhibitors are sparse (28, 29), this proposed interaction between calcineurin inhibitors and VEGF inhibition is supported by a number of studies which have reported higher-than-expected incidence of TMA when combining calcineurin inhibitors with mTOR inhibitors (30–33), as mTOR inhibitors as a class also have anti-VEGF properties (34). This observation has potential ramifications for patients taking calcineurin inhibitors in conjunction with VEGF inhibitors.

## Conclusion

This report outlined the first biopsy-proven case of TMA attributable to a VEGF inhibitor in a kidney transplant recipient, and the second case of biopsy-proven TMA attributable to the use of pazopanib. This case introduces the important consideration that combining calcineurin and VEGF inhibitors in transplant recipients may increase the likelihood of TMA-related events in this already susceptible population. This hypothesis is worthy of further investigation.

## References

[1] George JN, Nester CM. Syndromes of thrombotic microangiopathy. N Engl J Med. 2014;371(7):654–66. 10.1056/NEJMra1312353 25119611

[2] Brocklebank V, Wood KM, Kavanagh D. Thrombotic microangiopathy and the kidney. Clin J Am Soc Nephrol. 2018;13(2):300–17. 10.2215/CJN.00620117 29042465 PMC5967417

[3] Babaian RJ, Swanson DA. Serum haptoglobin: A nonspecific tumor marker for renal cell carcinoma. Southern Med J. 1982;75(11):1345–8. 10.1097/00007611-198211000-00010 7146964

[4] Del Vecchio SJ, Ellis RJ. Cabozantinib for the management of metastatic clear cell renal cell carcinoma. J Kidney Cancer VHL. 2018;5(4):1–5. 10.15586/jkcvhl.2018.109 PMC6175852 30319937

[5] La Manna G, Baraldi O, Corradetti V, Comai G. Cabozantinib-induced renal thrombotic microangiopathy. Nephrology. 2018;23(1):96–7. 10.1111/nep.13086 29250919 PMC6084320

[6] Vigneau C, Lorcy N, Dolley-Hitze T, Jouan F, Arlot-Bonnemains Y, Laguerre B, et al. All anti-vascular endothelial growth factor drugs can induce “pre-eclampsia-like syndrome”: A RARe study. Nephrol Dial Transplant. 2014;29(2):325–32. 10.1093/ndt/gft465 24302609

[7] Izzedine H, Escudier B, Lhomme C, Pautier P, Rouvier P, Gueutin V, et al. Kidney diseases associated with anti-vascular endothelial growth factor (VEGF): An 8-year observational study at a single center. Medicine. 2014;93(24):333–9. 10.1097/MD.0000000000000207 25500702 PMC4602430

[8] Al-Nouri ZL, Reese JA, Terrell DR, Vesely SK, George JN. Drug-induced thrombotic microangiopathy: A systematic review of published reports. Blood. 2015;125(4):616–8. 10.1182/blood-2014-11-611335 25414441 PMC4304106

[9] Eremina V, Jefferson JA, Kowalewska J, Hochster H, Haas M, Weisstuch J, et al. VEGF inhibition and renal thrombotic microangiopathy. N Engl J Med. 2008;358(11):1129–36. 10.1056/NEJMoa0707330 18337603 PMC3030578

[10] Estrada CC, Maldonado A, Mallipattu SK. Therapeutic inhibition of VEGF signaling and associated nephrotoxicities. J Am SocNephrol. 2019;30(2):187–200. 10.1681/ASN.2018080853 PMC6362621 30642877

[11] Qi WX, Lin F, Sun YJ, Tang LN, He AN, Yao Y, et al. Incidence and risk of hypertension with pazopanib in patients with cancer: A meta-analysis. Cancer Chemother Pharmacol. 2013;71(2):431–9. 10.1007/s00280-012-2025-5 23178953

[12] Bible KC, Suman VJ, Molina JR, Smallridge RC, Maples WJ, Menefee ME, et al. Efficacy of pazopanib in progressive, radioiodine-refractory, metastatic differentiated thyroid cancers: Results of a phase 2 consortium study. Lancet Oncol. 2010;11(10):962–72. 10.1016/S1470-2045(10)70203-5 20851682 PMC3107731

[13] Zhang ZF, Wang T, Liu LH, Guo HQ. Risks of proteinuria associated with vascular endothelial growth factor receptor tyrosine kinase inhibitors in cancer patients: A systematic review and meta-analysis. PloS ONE. 2014;9(3):e90135. 10.1371/journal.pone.0090135 24621598 PMC3951202

[14] Sternberg CN, Davis ID, Mardiak J, Szczylik C, Lee E, Wagstaff J, et al. Pazopanib in locally advanced or metastatic renal cell carcinoma: Results of a randomized phase III trial. J Clin Oncol. 2010;28(6):1061–8. 10.1200/JCO.2009.23.9764 20100962

[15] Sorich MJ, Rowland A, Kichenadasse G, Woodman RJ, Mangoni AA. Risk factors of proteinuria in renal cell carcinoma patients treated with VEGF inhibitors: A secondary analysis of pooled clinical trial data. Brit J Cancer. 2016;114(12):1313–7. 10.1038/bjc.2016.147 27228299 PMC4984472

[16] Kandula P, Agarwal R. Proteinuria and hypertension with tyrosine kinase inhibitors. Kidney Int. 2011;80(12):1271–7. 10.1038/ki.2011.288 21900879

[17] Novartis Pharmaceuticals Corporation. Votrient® [Internet]. [updated Aug 2020]. East Hanover, NJ: Novartis Pharmaceuticals Corporation. Available from: https://www.hcp.novartis.com/products/votrient/

[18] Négrier S, Pérol D, Bahleda R, Hollebecque A, Chatelut E, Boyle H, et al. Phase I dose-escalation study of pazopanib combined with bevacizumab in patients with metastatic renal cell carcinoma or other advanced tumors. BMC Cancer. 2017;17(1):547. 10.1186/s12885-017-3527-7 28810837 PMC5558713

[19] Maruyama K, Nakagawa N, Suzuki A, Kabara M, Matsuki M, Shindo M, et al. Pazopanib-induced endothelial injury with podocyte changes. Int Med. 2018;57(7):987–91. 10.2169/internalmedicine.9576-17 PMC5919858 29269661

[20] Syed U, Wahlberg KJ, Douce DR, Sprague JR. Thrombotic thrombocytopenic purpura associated with pazopanib. Case Rep Hematol. 2018;2018:4327904. 10.1155/2018/4327904 30057830 PMC6051109

[21] Reynolds JC, Agodoa LY, Yuan CM, Abbott KC. Thrombotic microangiopathy after renal transplantation in the United States. Am J Kidney Dis. 2003;42(5):1058–68. 10.1016/j.ajkd.2003.07.008 14582050

[22] Garg N, Rennke HG, Pavlakis M, Zandi-Nejad K. De novo thrombotic microangiopathy after kidney transplantation. Transplant Rev. 2018;32(1):58–68. 10.1016/j.trre.2017.10.001 29157988

[23] Ponticelli C. De novo thrombotic microangiopathy. An underrated complication of renal transplantation. Clin Nephrol. 2007;67(6):335–40. 10.5414/CNP67335 17598367

[24] Sartelet H, Toupance O, Lorenzato M, Fadel F, Noel LH, Lagonotte E, et al. Sirolimus-induced thrombotic microangiopathy is associated with decreased expression of vascular endothelial growth factor in kidneys. Am J Transplant. 2005;5(10):2441–7. 10.1111/j.1600-6143.2005.01047.x 16162193

[25] Izzedine H, Massard C, Soria JC. Unlikely association of nephrectomy post-mRCC with anti-VEGF-induced renal TMA. NDT Plus. 2011;4(1):78–9. 10.1093/ndtplus/sfq178 25984113 PMC4421633

[26] Person F, Rinschen MM, Brix SR, Wulf S, Noriega MLM, Fehrle W, et al. Bevacizumab-associated glomerular microangiopathy. Modern Pathol. 2019;32(5):684–700. 10.1038/s41379-018-0186-4 30552416

[27] Zuber J, Le Quintrec M, Morris H, Frémeaux-Bacchi V, Loirat C, Legendre C. Targeted strategies in the prevention and management of atypical HUS recurrence after kidney transplantation. Transplant Rev. 2013;27(4):117–25. 10.1016/j.trre.2013.07.003 23937869

[28] Müsri FY, Mutlu H, Eryılmaz MK, Salim DK, Cos¸kun H. Experience of bevacizumab in a patient with colorectal cancer after renal transplantation. J Cancer Res Ther. 2015;11(4):1018–20. 10.4103/0973-1482.168996 26881574

[29] Sorafenib/tacrolimus. React Wkly. 2019;1756(1):314. 10.1007/s40278-019-63193-9

[30] Cutler C, Henry NL, Magee C, Li S, Kim HT, Alyea E, et al. Sirolimus and thrombotic microangiopathy after allogeneic hematopoietic stem cell transplantation. Biol Blood Marrow Transplant. 2005;11(7):551–7. 10.1016/j.bbmt.2005.04.007 15983555

[31] Paramesh AS, Grosskreutz C, Florman SS, Gondolesi GE, Sharma S, Kaufman SS, et al. Thrombotic microangiopathy associated with combined sirolimus and tacrolimus immunosuppression after intestinal transplantation. Transplantation. 2004;77(1):129–31. 10.1097/01.TP.0000092522.36410.D0 14724447

[32] Shayani S, Palmer J, Stiller T, Liu X, Thomas SH, Khuu T, et al. Thrombotic microangiopathy associated with sirolimus level after allogeneic hematopoietic cell transplantation with tacrolimus/sirolimus-based graft-versus-host disease prophylaxis. Biol Blood Marrow Transplant. 2013;19(2):298–304. 10.1016/j.bbmt.2012.10.006 23078784 PMC3589900

[33] Langer RM, Van Buren CT, Katz SM, Kahan BD. De novo hemolytic uremic syndrome after kidney transplantation in patients treated with cyclosporine-sirolimus combination. Transplantation. 2002;73(5):756–60. 10.1097/00007890-200203150-00017 11907423

[34] Faes S, Santoro T, Demartines N, Dormond O. Evolving significance and future relevance of anti-angiogenic activity of mTOR inhibitors in cancer therapy. Cancers. 2017;9(11):152. 10.3390/cancers9110152 PMC5704170 29104248

